# High-Frequency EEG Variations in Children with Autism Spectrum Disorder during Human Faces Visualization

**DOI:** 10.1155/2017/3591914

**Published:** 2017-09-06

**Authors:** Celina A. Reis Paula, Camille Reategui, Bruna Karen de Sousa Costa, Caio Queiroz da Fonseca, Luana da Silva, Edgard Morya, Fabricio Lima Brasil

**Affiliations:** ^1^Edmond and Lily Safra International Institute of Neuroscience, Santos Dumont Institute, Rod. RN 160, Km 03, No. 3003, 59280-000 Macaiba, RN, Brazil; ^2^Anita Garibaldi Center for Education and Research in Health, Santos Dumont Institute, Rod. RN 160, Km 02, No. 2010, 59280-970 Macaiba, RN, Brazil; ^3^Electrical Engineering Department, Federal University of Campina Grande (UFCG), 882 Aprígio Veloso St, 58429-900 Campina Grande, PB, Brazil; ^4^Electrical Engineering Department, Federal University of Santa Maria (UFSM), 1000 Roraima Av., 97105-900 Santa Maria, RS, Brazil

## Abstract

Autism spectrum disorder (ASD) is a neuropsychiatric disorder characterized by the impairment in the social reciprocity, interaction/language, and behavior, with stereotypes and signs of sensory function deficits. Electroencephalography (EEG) is a well-established and noninvasive tool for neurophysiological characterization and monitoring of the brain electrical activity, able to identify abnormalities related to frequency range, connectivity, and lateralization of brain functions. This research aims to evidence quantitative differences in the frequency spectrum pattern between EEG signals of children with and without ASD during visualization of human faces in three different expressions: neutral, happy, and angry. Quantitative clinical evaluations, neuropsychological evaluation, and EEG of children with and without ASD were analyzed paired by age and gender. The results showed stronger activation in higher frequencies (above 30 Hz) in frontal, central, parietal, and occipital regions in the ASD group. This pattern of activation may correlate with developmental characteristics in the children with ASD.

## 1. Introduction

Autism spectrum disorder is a neurodevelopmental disorder with well-defined diagnostic criteria such as communications/social interaction and behavior deficits, with restricted and repetitive interests and activities [1, 2]. These social communication disturbances present a complex and heterogeneous pattern and for that reason fit in a spectrum and may present various patterns of severity in symptoms or evolution profile. The initial publications about the autism spectrum disorder were only in the form of case reports [3, 4]. In 1966, the first epidemiological study suggested the occurrence of 4.5 children with ASD per 10,000 inhabitants in the age range of 8 to 10 [5]. However, further studies realized in Europe, United States, Canada, and Japan suggest an increase in this incidence, with values of 10, 30, or even 60 cases per 10,000 inhabitants [6]. Nowadays, the ASD seems to affect approximately 1 in 68 children, most common among boys [7]. There is a suggestion of increased incidence and prevalence of ASD worldwide [8]. This increase is probably due to the changes in the diagnostic criteria [9], emergence and development of diagnostics, early intervention services, and more direct approaches related to the diagnosis of the disorder [10].

The ASD diagnostics are realized according to the observation of initial symptoms in early childhood with impairments in their daily functionality [2], neurodevelopment, children's behavioral characteristics, and objective clinical analysis. The clinical signs should fulfill the diagnostic criteria described in the Diagnostic and Statistical Manual for Autism (DSM-V). Questionnaires, checklists, and diagnostic scales support the evaluation and confirm the diagnosis [11]. Children with ASD can present a heterogeneous clinical picture, in which the behavioral symptoms prevail. Such facts boost a search for biological markers of the disorder using different tools, for example, the electroencephalogram (EEG), the Eye-Tracker, the Functional and Structural Magnetic Resonance Imaging (fMRI or MRI), Positron Emission Tomography (PET), and Computerized Tomography (CT) based on emission of single photon (SPECT) [12]. These tools have been increasingly exploited in scientific research.

Abnormal EEG activity of the epileptic type occurs in 30% of the cases of ASD, even without epileptic seizures [13]. Paroxysmal discharges and slow focal activity were registered in the temporal region in EEG of patients with the disorder, especially those with developmental regression [14]. Although EEG can provide important information about brain function during resting and stimulation, the qualitative visual signal analysis of time domain seems to be insufficient to consider a pathognomonic pattern for the ASD [15]. Techniques for quantitative analysis, such as the Fourier Transform, favor a more detailed frequency analysis by bandwidth and its characteristics. EEG analysis of children with ASD shows differences in brain electrical signals compared to children without ASD [5, 16]. A reduction in the power spectrum in the alpha bandwidth (4–8 Hz) was previously observed in EEGs of children with ASD during rest [14]. Also, hemispheric asymmetry of activity has been shown, such as greater activity in the left frontal lobe when observing happy facial expressions with smiles [17] and decreased activity in the same region when observing facial expressions of fear [18]. It was also observed that a theta power spectrum in the frontal midline (Fm) is related to emotional states. Sammler and colleagues proposed that pleasant emotions (opposed to unpleasant) are related to the increase in the theta power density in the Fm [19]. Thus, in light of previous studies and considering the behavioral component in the diagnosis of ASD, it is expected that the use of the EEG to access neural activities elicited by social stimuli has the potential of providing a quantitative analysis of impairment in social interaction activities of this group [20].

Neuropsychological patterns verified in ASD might suggest the involvement of other brain regions. The difficulty in maintaining attentional focus or the behavior of paying attention to a face or object details instead of the whole picture might be related to difficulty in shared attention and in the executive function, capabilities that involve the frontal region.

The current study aims to show quantitative differences in the frequency spectrum pattern between the EEG of children with and without ASD before and during the observation of human faces. We believe that finding these differences can lead to a better understanding of how these children could potentially be better stimulated and taught using their respective preference of human faces or figure faces. We hypothesize that social interaction impairment present in children with ASD, when compared with children without ASD, can occur due to a deficit in the visual processing of human faces. In addition, we also hypothesize that the brain's electrical activity presents a different quantitative pattern in the power spectrum in the bandwidths during the observation of stimulus such as human faces with different emotion expressions.

## 2. Materials and Methods

### 2.1. Participants

This research was authorized by the Ethics Committee of the Federal University of Rio Grande do Norte (CAAE 46207015.0.0000.5537). The consent form was read and explained to the parents, and after their agreement, they signed the consent form. EEG data were recorded at the Anita Garibaldi Center for Education and Research in Health (CEPS). Sixteen children participated in the study: eight with ASD and 8 without ASD. All of them were from the same metropolitan region and were paired by age (Table 1). The age of the participants varied from 5 to 12 years (*M* = 8.44, SD = 2.24), and only two (one of each group) were female. The inclusion criteria for the group with ASD were the presence of the diagnostics realized by a neuropediatrician after neuropsychological evaluation. For the children of the control group, it was necessary to test for intellectual disability with the ASD diagnostics, also by the same team. Children with epileptic seizures in the last 3 years did not participate in the research. Subjects who did not cooperate to perform the exam also were excluded and respective data were not included in the analysis.

The ASD diagnostics were realized by a neuropediatric physician, considering the fulfillment of the diagnostics criteria of the DSM-V, the neuropsychological evaluation with the childhood autism rating scale (CARS), an IQ test, and language evaluation.

### 2.2. Experimental Setup

#### 2.2.1. EEG

EEG-1200 (Neurofax, Nihon Kohden, Tokyo, Japan) was used for noninvasive brain electrophysiological signal acquisition (sampling rate 1.000 Hz) with 22 electrodes (Ag/AgCl disk electrode, 10/20 distribution with ear lobes ground) positioned with previous scalp preparation (cleaned with neutral soap, dry and no hair creams or hair products, and impedance lower than 5 kΩ). EEG data were recorded and synchronized (StimTracker ST-100, Cedrus, USA) with visual stimulation and eye-tracking.

#### 2.2.2. Visual Stimuli

**Figure 1:**
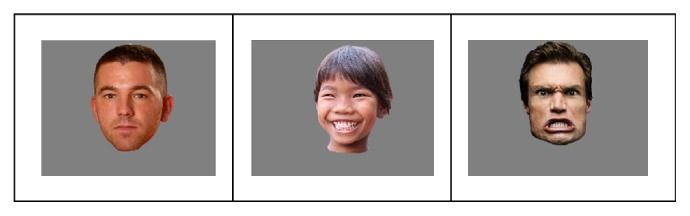
Images showing human faces with neutral, happy, and angry expressions.

Visual stimuli with 30 human faces were presented on a grey background (Figure 1). The faces were paired by expression and classified into three groups: 10 neutral, 10 happy, and 10 angry.

All stimuli were size dimensioned and standardized to keep equivalent distances between the eyes, mouth, and nose in a central square area of the 17′′ LCD screen (100 Hz, Samsung) 60 cm from the participant's eyes. E-Prime® 2.0 software (Psychology Software Tools, Inc., USA) presented in a sequence (happy-neutral-angry faces), interspersed by a fixation point in the center of the screen. Each face was presented for 3 seconds with 0.5 to 1.0 s of interval controlled by Mangold Vision 3.9 (Mangold International GmbH, Germany) software in programmed sequence and time.

#### 2.2.3. Eye-Tracking

An Eye-Tracker (Eye-Tech TM3 60 Hz, Mesa, USA) was positioned under the screen to ensure the participants were looking at fixation point and visual stimuli during the task.

#### 2.2.4. Task

Participants sat comfortably in a quiet dimmed room 60 cm from the LCD screen with EEG electrodes. They were instructed to keep the eyes at the fixation point and look at the image during the presentation. After eye-tracking calibration, EEG recording started two minutes before the visual stimulation.

### 2.3. Data Preprocessing

EEG data underwent preprocessing with a custom MATLAB (Mathworks, USA) script, EDF Browser (© Copyright 2017 Teunis van Beelen), and Python (Python Software Foundation).

#### 2.3.1. EDF Browser

EDF Browser converted raw EEG data to ASCII format compatible with Python and MATLAB. The epochs to be analyzed were correctly separated according to the stimuli marker registered during the experiment in order to organize files. Thus, for each subject, three files were generated containing, respectively, all epochs of happy, neutral, and angry faces, for all EEG channels. These files were then processed by Python Programming Language.

#### 2.3.2. Python Programming Language

Python libraries for data manipulation and analysis were used to organize sets of trials with all subjects of each group and separate channels. This resulted in.csv files with raw EEG data of subjects for each type of stimuli and for each channel. Thereafter, these files were processed by MATLAB.

#### 2.3.3. MATLAB

A MATLAB code designed a 6th-order bandpass Butterworth filter with a lower cutoff frequency of 1 Hz and a higher cutoff frequency of 100 Hz. A Notch filter removed the frequency component of the electrical grid (58–62 Hz). Moreover, a technique for detecting spectral perturbation related to the event (ERSP) was implemented. The ERSP consists of a tool to observe the variations on local field potentials related to the event by calculating the mean and the standard deviation of the EEG signal in order to normalize the signal prior to the event. Each epoch has −500 ms before stimulus presentation and +3.500 ms. Spectral features related to the event were calculated for this time window. After that, the same procedure was done in the event epochs, where they were analyzed with the ERSP tool in order to calculate the variation or spectral perturbation related to the event occurrence.

## 3. Data Analysis and Results

### 3.1. Clinical Results

Group ASD presented verbal patterns, with 37.5% able to speak few words and sentences, but not enough to maintain a dialogue. Control group also presented verbal patterns, with 100% able to speak few words, sentences, and adequate conversation. All children were attending school, 50% of group ASD were literate, and 50% were in the presyllabic stage. In the control group, only one subject (4 years old) was in the presyllabic stage and the others (87%) were literate.

The ASD's diagnostic age was 4.77 years (SD = 2.30). The ASD parents' age was 40.37 ± 4.59 years and the control group parent's age was 34.62 ± 8.17 years. The predominant parent's education level was college (43.8%) for the ASD group and high-school (56.3%) for the control group. The average income for both groups was between 1 and 5 minimum salaries (75%).

### 3.2. EEG Power Spectral Density

EEG Power Density Spectrograms were generated using MATLAB for the mean of ASD and control group. Differences between ASD and control groups were observed in power spectrum parameters, with stronger activation for Gamma band (above 30 Hz), and along frontal, central, parietal, and occipital electrodes.

For the ASD group, the major activation was verified in the Fp and F electrodes for frequencies above 20 Hz, in the parietal and central electrodes for frequencies between 40 and 50 Hz, and in the occipital electrodes for frequencies above 40 Hz. In a lower incidence, there was also an increase of slow activity (below 8 Hz) in the frontal, parietal, and occipital regions. In general, the major activation occurred in the left brain hemisphere for the ASD group.

**Figure 2:**
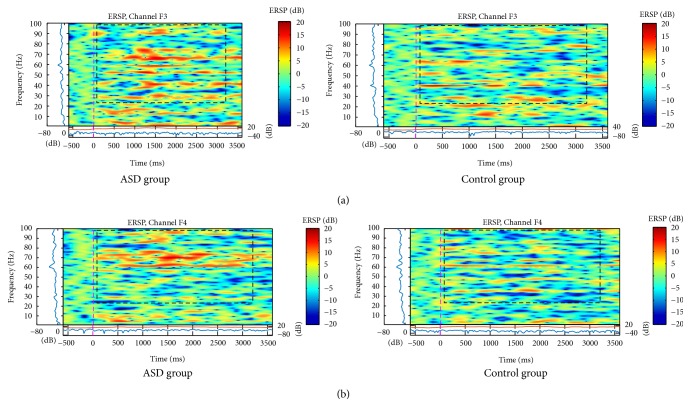
ERSP of (a) F3 and (b) F4 electrodes for the total mean of samples for angry expression signal in an interval of 3 seconds (plus a basal activity of 500 ms before and after an event) for ASD and control groups that showed significant variations or spectral perturbations. The ERSP image in the upper panel presents the ERSP (Event-Related Spectral Perturbation) data in dB, with mean baseline spectral power subtracted at each time in the epoch. The upper left marginal panel presents mean spectral power during the baseline period (blue). The marginal panel under the ERSP image shows the maximum (red) and minimum (blue) ERSP values relative to baseline power for each frequency.

Graphics were generated with the mean of each group according to the type of stimulus (neutral, happy, and angry expressions) and only graphics that showed differences between the ASD and control groups were presented. In this case, the distinctness of the evaluation responses was confirmed in the F3/F4, C3/C4, P3/P4, and O1/O2 electrodes. In frontal electrodes, the activation was bigger in the ASD group and mainly in higher frequencies (above 30 Hz), but it was also increased in the theta and delta bands for angry faces, as shown in Figures 2(a) and 2(b).

**Figure 3:**
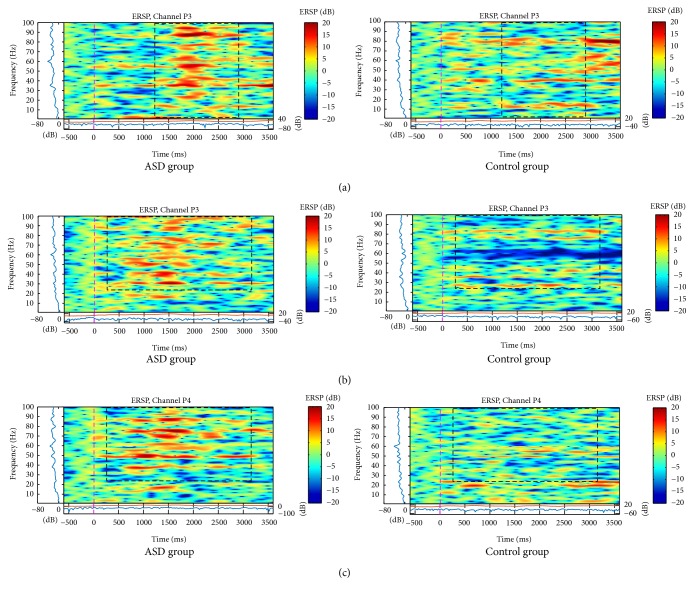
ERSP of P3 (a, b) and P4 (c) electrodes for total mean of samples of the neutral (a), and angry (b), and (c) faces signals in interval of 3 seconds (plus a baseline activity of 500 ms before the event) for ASD and control groups that showed significant spectral perturbations. In ERSP image, the upper panel presents the ERSP (Event-Related Spectral Perturbation) data in dB, with mean baseline spectral power (in dB) subtracted at each time in the epoch. Upper left marginal panel presents the mean spectral power during the baseline period (blue). Marginal panel under the ERSP image shows the maximum (red) and minimum (blue) ERSP values relative to baseline power at each frequency.

In parietal electrodes, the differences between the groups were also verified. ASD group presented more activation in slow frequencies (below 5 Hz) for neutral faces on electrode P3 and higher frequencies (above 30 Hz) for neutral faces as well as for angry faces, having a symmetric pattern only for the latter (Figure 3). Analysis of variance showed a main effect for Gamma band on electrodes P3, *F*(1,1118) = 9.55, *p* < .000, and P4, *F*(1,1118) = 6.20, *p* < .000. Post hoc independent-samples *t*-test indicated that scores for electrode P3 were significantly higher for the ASD group (*M* = .255, SD = .341) than for the control group (*M* = .071, SD .200), *t*(1118) = 11.1, *p* < .001.

**Figure 4:**
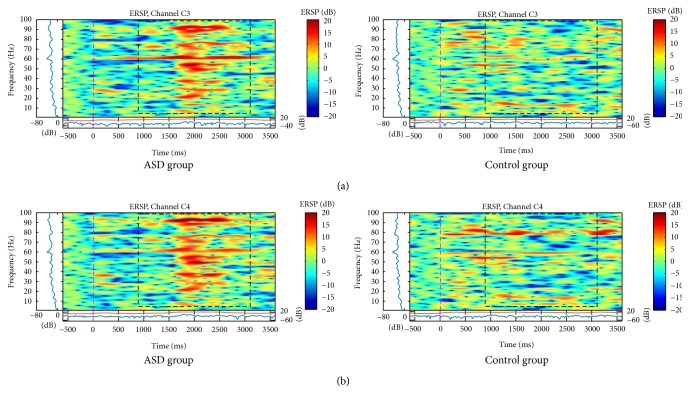
ERSP of (a) C3 and (b) C4 electrodes for the total mean of samples of the neutral faces signals in the interval of 3 seconds (plus a basal activity of 500 ms before the event) for the ASD and control groups that showed significant spectral perturbations. In the ERSP image, the upper panel presents the ERSP (Event-Related Spectral Perturbation) data in dB, with mean baseline spectral power (in dB) subtracted at each time in the epoch. The upper left marginal panel presents the mean spectral power during the baseline period (blue). The marginal panel under the ERSP image shows the maximum (red) and minimum (blue) ERSP values relative to baseline power at each frequency.

**Figure 5:**
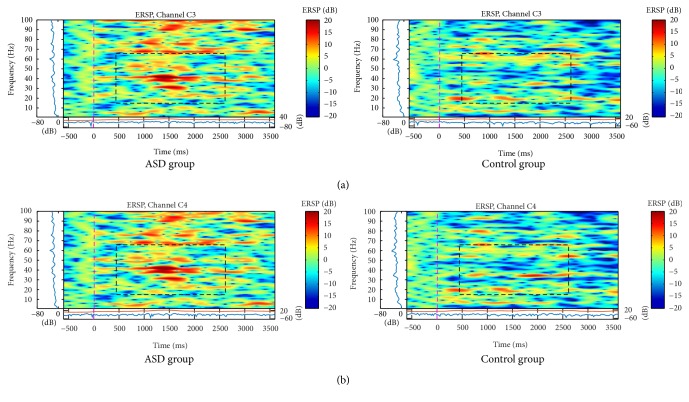
ERSP of (a) C3 and (b) C4 electrodes for the total mean of samples of the angry faces signals in the interval of 3 seconds (plus a basal activity of 500 ms before the event) for the ASD and control groups that showed significant variations or spectral perturbations. In the ERSP image, the upper panel presents the ERSP (Event-Related Spectral Perturbation) data in dB, with mean baseline spectral power (in dB) subtracted at each time in the epoch. The upper left marginal panel presents the mean spectral power during the baseline period (blue). The marginal panel under the ERSP image shows the maximum (red) and minimum (blue) ERSP values relative to baseline power at each frequency.

The C3 and C4 electrodes showed higher bilateral activation in all frequency bands in the ASD group to the stimulus of neutral (Figure 4) and angry (Figure 5) faces. Analysis of variance showed a main effect for Gamma band on electrodes C3, *F*(1,1118) = 8.36, *p* < .000, and C4, *F*(1,1118) = 75.1, *p* < .000. Post hoc independent-samples *t*-test indicated that scores for electrode C3 were significantly higher for the ASD group (*M* = .287, SD = .340) than for the control group (*M* = .114, SD .266), *t*(1118) = 9.47, *p* < .001, and the same pattern was found for electrode C4, where the ASD group were higher (*M* = .255, SD .327) than for the control group (*M* = .103, SD = .253), *t*(1118) = 8.67, *p* < .001.

The activation was similar for parietal and central regions on neutral faces (similarity just on the left side) and mainly for angry faces (bilateral similarity). Neutral faces showed an increase of spectral power between 10 and 100 Hz, mostly at 2 seconds, in C3, C4 (Figure 4), and P3 channels (Figure 3(a)), but only for ASD group.

For angry face stimulus, spectral power was higher in higher frequencies (between 20 and 50 Hz), on C3 and C4 channels, at 1.5 seconds (Figure 5). The P3 (Figure 3(b)) and P4 (Figure 3(c)) channels similarly showed higher power in higher bands (above 60 Hz).

**Figure 6:**
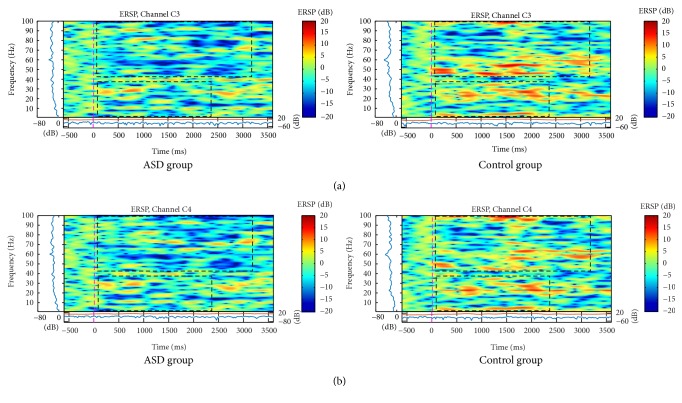
ERSP of (a) C3 and (b) C4 electrodes for the total mean of samples of the happy faces signals in the interval of 3 seconds (plus a basal activity of 500 ms before the event) for the ASD and control groups that showed significant variations or spectral perturbations. In the ERSP image the upper panel presents the ERSP (Event-Related Spectral Perturbation) data in dB, with mean baseline spectral power (in dB) subtracted at each time in the epoch. The upper left marginal panel presents the mean spectral power during the baseline period (blue). The marginal panel under the ERSP image shows the maximum (red) and minimum (blue) ERSP values relative to baseline power at each frequency.

For the happy face stimulus, the C3 and C4 channels of the control group presented activation in frequencies under 8 Hz from the beginning of the stimuli till 2 seconds later (Figure 6). Simultaneously, there was an activation in higher frequencies (above 30 Hz). However, in ASD group, there was desynchronization mainly in higher frequencies (above 30 Hz) until the end of the stimulus.

**Figure 7:**
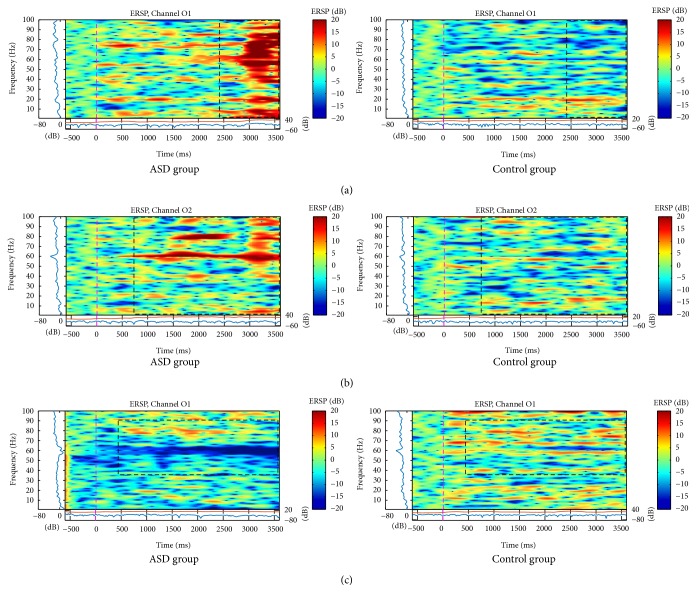
ERSP of (a) and (c) O1 and (b) O2 electrodes for the total mean of the samples of the happy (a) and (b) and neutral (c) faces signals in the interval of 3 seconds (plus a basal activity of 500 ms before the event) for the ASD and control groups that showed significant spectral perturbations. In the ERSP image, the upper panel presents the ERSP (Event-Related Spectral Perturbation) data in dB, with mean baseline spectral power (in dB) subtracted at each time in the epoch. The upper left marginal panel presents the mean spectral power during the baseline period (blue). The marginal panel under the ERSP image shows the maximum (red) and minimum (blue) ERSP values relative to baseline power at each frequency.

**Figure 8:**
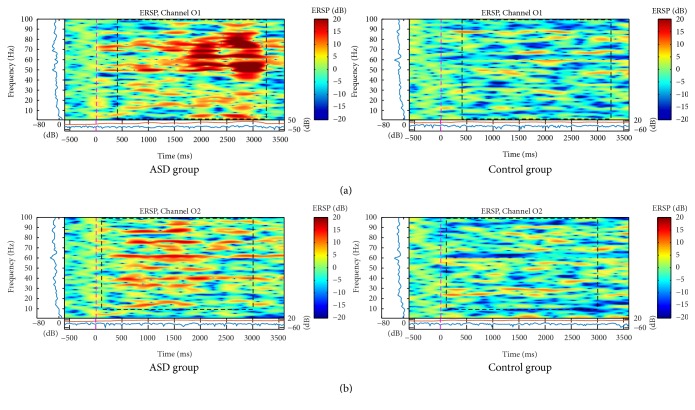
ERSP of (a) O1 and (b) O2 electrodes for the total mean of the samples of the angry faces signals in the interval of 3 seconds (plus a basal activity of 500 ms before the event) for the ASD and control groups that showed significant variations or spectral perturbations. In the ERSP image, the upper panel presents the ERSP (Event-Related Spectral Perturbation) data in dB, with mean baseline spectral power (in dB) subtracted at each time in the epoch. The upper left marginal panel presents the mean spectral power during the baseline period (blue). The marginal panel under the ERSP image shows the maximum (red) and minimum (blue) ERSP values relative to baseline power at each frequency.

Differences occurred in occipital electrodes in the ASD group for the three types of faces, and there was a bilateral desynchronization, but mainly in the left hemisphere for neutral faces (Figure 7(c)). Also, on the left hemisphere, there were differences between happy and angry faces due to a significant increase in spectral power in all frequency bands, mainly the higher bands right after the happy pattern stimulus (Figures 7(a) and 7(b)) and during the angry pattern stimulus (Figure 8). In this case, significant activation occurred to the right hemisphere also in the ASD group before happy faces and there was no asymmetry in the control group (Figures 7 and 8).

## 4. Discussion

In this study, EEG analyses in children with ASD diagnostics paired with children without ASD were compared during the observation of faces with neutral, happy, and angry expression. Children with ASD presented stronger power spectrum in higher frequencies than the control group for some brain areas. Differences were more evident in occipital and center-parietal regions. Central regions showed a similar pattern to parietal, with same power activation in the same time that the stimulus was presented. Given the clinical evidence of an emotional, cognitive, and behavioral deregulation [21], one of the possible explanations is a perturbation in brain function with stronger or weaker connectivity between areas like the amygdala and prefrontal ventrolateral cortex and orbitofrontal cortex [21].

Developmental psychology suggests that children imitate facial gestures from an early age. This premature imitation might be related to a direct connection from a visual input to a specific motor output [22]. This function is related to the systems of mirror neurons [23]. Failures in this system may be related to the social cognition deficits of ASD. This system suggests a strong relationship between action and intention recognition with social cognition, since it seems to regulate premotor cortex during observation action [24].

It is believed that mirror neurons form a system localized in the inferior parietal lobe, inferior frontal gyrus, superior temporal sulcus, and parietal-frontal lobe. Mirror neurons can be activated by visual stimulus. It was observed that, for visual stimuli indicating action, children with ASD present stronger activation of primary motor areas when compared to activation in the supplementary motor area [25].

Formation of the local network is needed for typical development in childhood and, after that, distribution of neural network in the teenage years and adult phase [26]. ASD children seem to have an atypical organization of the primary motor cortex, resulting in a subconnectivity with weak and short functional reach [26]. These subnetworks might generate execution loss of gestures linked to communication with consequent influence on social behavior [26].

Children with ASD can present differences in brain activities in visual-spatial processing related to object recognition (occipital, temporal, and ventral) and localization of objects in space (parietal, temporal, and dorsal). Communication failures between the dorsal and ventral pathways can harm the visual processing [27] and a lesion in these areas can lead to visual negligence and spatial distortions of body movements [27]. Various neurophysiological studies [7, 28–30] have tried to correlate these clinical symptoms through the demonstration of deficiencies or functional abnormalities of neural networks.

The findings of this study can be correlated to the clinical signs of children with ASD. The hypothesis is that, in ASD children, a deficit in facial expression processing will occur, with consequent failure in the storage for posterior access. The primary visual and primary motor areas are well activated, with some influence of decoding of the parietal lobe. Because of that, a bigger power spectrum might have occurred practically of the same pattern of central and parietal regions. It is believed that, in C3 and C4 electrodes during the stimuli of neutral and angry face, the presence of excessive fast rhythm in the ASD group when compared to the control group is probably due to the bottom-up activation. These failures of connectivity promote a mirror neurons system behavior similarly to the imitation of immature children that presents the direct conversion of the stimulus input (primary visual cortex) and motor output (motor cortex). In contrast, children with ASD have visual tracking patterns with greater fixation in regions important for emotional expressiveness (such as eyes and mouth) when exposed to happy face stimuli [28]. This behavior may help a more emotional maturity processing and therefore, a cerebral hypoactivation in C3 and C4 for fast frequency bands in this group in relation to the control was observed in this study. Similarly, there is a flaw in the rendering of faces with emotion mainly for neutral and angry expressions.

Another explanation for the motor deficits of child with ASD is the failure in visual and motor circuitry. Stereotyped and repetitive behaviors are reported in 64% of the ASD cases and the child realizes the inadequate movements mainly in the attempt of sensory regulation as a mechanism of an organization [31]. In ASD, proprioceptive prejudice associated with reception failure and visual stimuli processing harms motor learning and contributes to the motor behavior and social inappropriateness [32], facilitating the presence of the stereotyped and repetitive behavior. The sensory processing disorder (hypo- or hyperresponsive) may be related to the origin of the functional limitations of the child [33]. Also, there might be some prejudice in motor planning because of the inappropriate visual processing in the ASD. Knowing that the motor and sensory systems cooperate with each other [34], the joint failure of these systems can cause motor dysfunction. A second possible explanation for this study having verified more activation of fast frequencies in the center region is neural plasticity. In children with ASD, failure in sensory/visual processing and in planning together can promote differentiated motor behavior that might occur due to the activation of groups of neurons of primary function without regulation. ASD children might have a failure in the visual processing because of a modification in the communication between dorsal and ventral pathways, which are mediated by connections with the frontal cortex [27]. The failure of the visual processing can promote more activation in the primary visual area.

In this study, a high activation in the occipital areas was found. Bigger activation in O1 and O2 that occurred when happy and angry face group were presented might be due to stronger activation of the primary visual cortex relative to the control group. It is known that in ASD there are failures in visual perception and that it seems to cause more fixation to parts of the stimulus relative to the whole, and also there are erratic visualization patterns [35]. This more intense focal fixation and probable prejudice of face processing can justify the observed pattern. Maybe in the neutral face, the pattern did not occur because there were no “distractors” such as the muscular contraction that occurs in other expressions which attract more focal attention.

## 5. Conclusion

The analysis of the power spectrum in children with ASD during visual stimulus of happy, neutral, and angry faces demonstrated an increase of power in higher frequencies (above 30 Hz) in the ASD group in frontal, occipital, and center-parietal areas when compared to control group. More studies are needed to better understand these differences.

## Figures and Tables

**Table 1:** Participants demographics.

Experimental group (ASD)					Control group			
	Age (yrs)	Gender	Language structure	Age at diagnosis (yrs)		Age	Gender	Language structure
Subject 1	10	Male	Sentences	5	Subject 9	10	Male	Verbal fluency
Subject 2	12	Female	Phrases	5	Subject 10	12	Female	Verbal fluency
Subject 3	6	Male	Word	2	Subject 11	6	Male	Verbal fluency
Subject 4	7	Male	Sentences	3	Subject 12	7	Male	Verbal fluency
Subject 5	8	Male	Sentences	4	Subject 13	8	Male	Verbal fluency
Subject 6	9	Male	Word	4	Subject 14	9	Male	Verbal fluency
Subject 7	5	Male	Sentences	3	Subject 15	5	Male	Verbal fluency
Subject 8	11	Male	Sentences	4	Subject 16	11	Male	Verbal fluency
